# Ectopic parathyroid adenoma posterior to the trachea in the superior mediastinum causing refractory primary hyperparathyroidism: a case report

**DOI:** 10.3389/fonc.2026.1857424

**Published:** 2026-05-20

**Authors:** Li Yang, Hua Zhang, Jun Huang, Bei Wang, Minghua Wang, Jie Yuan

**Affiliations:** 1 Department of Clinical Laboratory Medicine, Hubei Provincial Clinical Research Center for Umbilical Cord Blood Hematopoietic Stem Cells, Taihe Hospital, Hubei University of Medicine, Shiyan, Hubei, China; 2 Department of Breast, Thyroid and Vascular Surgery, Taihe Hospital, Hubei University of Medicine, Shiyan, Hubei, China

**Keywords:** ectopic parathyroid tumor, missed diagnosis, primary hyperparathyroidism, superior mediastinum, surgical procedure

## Abstract

Primary hyperparathyroidism (PHPT) caused by ectopic parathyroid adenomas in the superior mediastinum is rare and often leads to surgical failure. We report a case of severe PHPT in a 50-year-old female presenting with lower limb weakness and bone pain. Laboratory tests revealed marked hypercalcemia (serum calcium 3.48 mmol/L) and extremely elevated parathyroid hormone (PTH) levels (1920 pg/mL). Preoperative imaging, including neck ultrasound and 99mTc-MIBI SPECT/CT, precisely localized the ectopic adenoma to the superior mediastinum at the T1 vertebral level, posterior to the trachea, rather than in conventional parathyroid regions. The patient underwent successful complete resection of the tumor via meticulous neck and superior mediastinal exploration. Postoperatively, PTH and serum calcium levels normalized rapidly. In patients with biochemically confirmed PHPT and negative imaging in classical locations, the value of accurate preoperative imaging is underscored by this case as is intraoperative imaging guided exploration.

## 1 Introduction

Primary hyperparathyroidism (PHPT) is a common endocrine disorder most frequently caused by a parathyroid adenoma (1). Approximately 85% of adenomas are located in the normal anatomical region posterior to the thyroid gland. However, 1–5% occur in ectopic locations, such as within the thyroid parenchyma, retroesophageal space, carotid sheath, or mediastinum (2). Ectopic parathyroid adenomas often pose challenges for preoperative localization and can lead to surgical failure (3). This report presents a case of an ectopic parathyroid adenoma located posterior to the trachea in the superior mediastinum and discusses key diagnostic and management points based on a literature review.

## 2 Case presentation

A 50-year-old female was admitted to our hospital in November 2025 with a two-year history of progressive lower limb weakness and pain. Two years prior, laboratory tests at another hospital revealed elevated serum calcium (exact value unknown) and elevated parathyroid hormone (PTH), leading to a diagnosis of hyperparathyroidism. Medical treatment (details unknown) was ineffective. The patient has no family history of related conditions. She was referred to our hospital for surgical evaluation.

On admission, physical examination showed stable vital signs. No palpable masses were detected in the neck, and the thyroid gland was not enlarged. Limb muscle strength was grade IV, without significant tenderness.

### 2.1 Laboratory findings

Serum calcium was 3.48 mmol/L (reference range: 2.11–2.52 mmol/L), serum phosphorus was 0.63 mmol/L (reference range: 0.85–1.51 mmol/L), and PTH was 1920 pg/mL (reference range: 15–65 pg/mL). Thyroid function tests were normal.

### 2.2 Imaging studies

1. Thyroid and parathyroid color Doppler ultrasound: Multiple hypoechoic nodules were found in the left thyroid lobe (largest: 45×32×25 mm, TI-RADS category III) and right thyroid lobe (largest: 13×13×12 mm, TI-RADS category III). No abnormalities were detected in the conventional parathyroid regions. A hypoechoic nodule measuring approximately 32×22×15 mm was identified inferior and deep to the right lower pole of the thyroid, in the supraclavicular fossa, suggestive of parathyroid origin (**Figure 1**).
2. 99mTc-MIBI SPECT/CT: A nodule posterior to the trachea at the T1 vertebral level, inferior to the right thyroid lobe, showed significant radiotracer uptake on both early and delayed imaging, highly suggestive of a parathyroid adenoma or hyperplasia (**Figure 2**). A heterogeneous density mass in the left thyroid lobe also showed MIBI uptake, warranting biopsy; nodules in the right lobe showed no uptake.
3. Chest and neck CT: Generalized osteoporosis with multiple cystic changes (brown tumors) was observed, consistent with severe hyperparathyroid bone disease (**Figure 3A**). Multiple thyroid masses and a superior mediastinal mass were also noted (**Figure 3B**).

**Figure 1:**
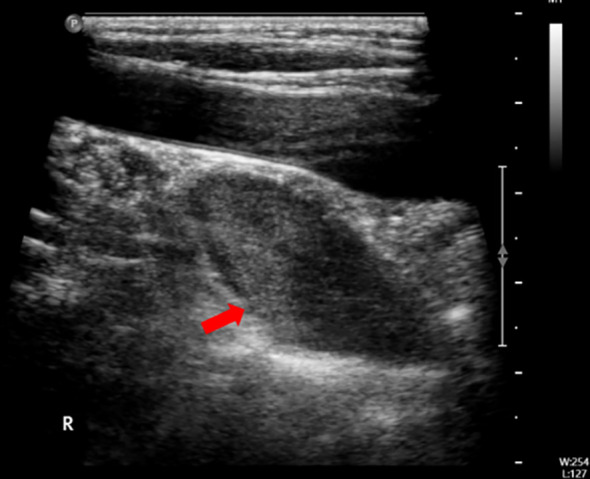
Preoperative neck ultrasound showing a hypoechoic nodule inferior to the right lower pole of the thyroid gland (arrow).

**Figure 2:**
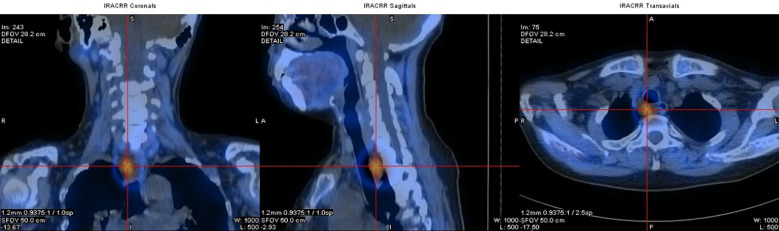
Parathyroid MIBI scan showing a focus of intense radiotracer uptake inferior to the thoracic inlet, anterior to the T1 vertebra.

**Figure 3:**
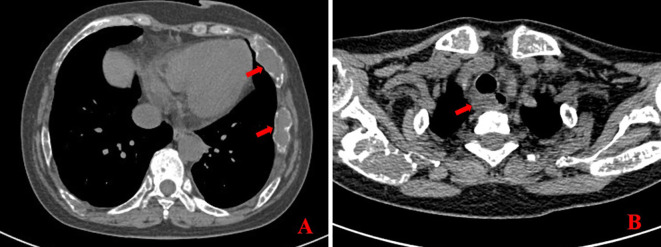
Chest and neck CT scan showing the lesions. **(A)** multiple irregular areas of bone destruction (brown tumors) are visible in the ribs (arrow). **(B)** the ectopic parathyroid tumor located at the level of the T1 vertebra, posterior and to the right of the trachea (arrow).

### 2.3 Treatment and surgical procedure

Based on the findings of hypercalcemia, elevated PTH, bone destruction, and imaging, the clinical diagnoses were: (1) primary hyperparathyroidism; (2) parathyroid adenoma; (3) bilateral thyroid nodules; and (4) hyperparathyroid bone disease.

Surgery was performed under general anesthesia on November 24, 2025. Bilateral subtotal thyroidectomy was performed first. Intraoperative frozen section of the thyroid nodules revealed “follicular neoplasm, benign or malignant status to be determined by paraffin section”. Subsequently, focused exploration for parathyroid tissue was conducted. Exploration of the bilateral dorsal aspects of the thyroid, tracheoesophageal grooves, and carotid sheaths—common parathyroid locations—revealed no enlarged parathyroid tissue. Guided by preoperative SPECT/CT images, exploration was directed posterior to the right recurrent laryngeal nerve and deep within the tracheoesophageal groove. After fully exposing and protecting the right recurrent laryngeal nerve, dissection proceeded toward the thoracic inlet and superior mediastinum. A firm, oval mass approximately 30×25×20 mm was identified anterior to the T1 vertebra and posterior to the right side of the trachea. It was anteriorly adjacent to the recurrent laryngeal nerve, posteriorly adherent to the prevertebral fascia, and its superior pole was near the bifurcation of the common carotid and subclavian arteries. The mass was carefully dissected and completely excised. Intraoperative frozen section confirmed “parathyroid tumor”.

### 2.4 Postoperative pathology and follow-up

Paraffin section pathology:

1. (Right neck mass) Parathyroid adenoma, mitotic figures <1/HPF, with focal fibrous tissue and compressed normal parathyroid tissue at the periphery. Immunohistochemistry: PTH (+), CgA (+), Syn (+), Rb (+), CK(P) (+), Ki-67 (approximately 1%).
2. (Bilateral thyroid masses) Thyroid follicular adenoma.

On postoperative day 1, serum calcium was 1.94 mmol/L, serum phosphorus 0.67 mmol/L, and PTH 38.7 pg/mL. The patient developed perioral and extremity numbness due to transient hypocalcemia, which resolved with intravenous and oral calcium and calcitriol supplementation. Recovery was uneventful, with no hoarseness, choking, or pneumothorax. One week postoperatively, PTH and serum calcium levels remained within normal ranges.

## 3 Discussion

PHPT refers to an excessive secretion of parathyroid hormone because of a parathyroid pathology which leads to disturbances of calcium, phosphorus and bone metabolism (4). The single most common type of parathyroid tumor is adenoma, which accounts for approximately 85% of cases. Hyperplasia accounts for about 10%, while adenocarcinoma accounts for <1%. The clinical manifestations include hypercalcemia, skeletal lesions (e.g. osteitis fibrosa cystica, osteoporosis, bone pain, pathological fractures), urinary stones, and neuropsychiatric disorders (5). This patient presented with severe bone pain and weakness with significant destruction of the bones on imaging. Serum calcium and PTH were markedly elevated. This was considered severe PHPT with classic bone disease.

### 3.1 Embryological foundation and frequent ectopic sites

The third and fourth pharyngeal pouches give rise to the parathyroid glands. The superior parathyroids (from the fourth pouch) are usually located symmetrically on the dorsum of the middle to upper thyroid gland. The inferior parathyroids migrate along with the thymus (which comes from the third pouch) and this results in greater positional variability (6). Ectopic sites include cervical sites (thyroid parenchyma, carotid sheath, retroesophageal, and retropharyngeal) (7, 8); thymus sites (most common, thymic tongue, anterior superior mediastinal thymus) (9); other mediastinal sites (aortopulmonary window, para-aortic, deep tracheoesophageal groove, retroesophageal, even pericardial) (10); rare sites (mandibular angle, parotid gland, base of skull) (11). The lesion in this case was found at the T1 vertebral level, posterior and right of the trachea in the superior mediastinum, a rare deep ectopia over the migration path of an inferior parathyroid gland.

### 3.2 Preoperative localization strategy

The success of the surgical procedure depends on accurate preoperative localization. Conventional neck ultrasound has high sensitivity for typical locations but is poor at detecting ectopic lesions, especially mediastinal ones, due to interference from bone, air, and depth (12). Ultrasound is used to identify a hypoechoic nodule inferior to the lower pole of the thyroid in our case. The most preferable method used for localization of ectopic or recurrent parathyroid lesions is the 99mTc-MIBI dual-phase scintigraphy with SPECT/CT (13). MIBI uptake by hyperfunctioning parathyroid tissues, CT provides good anatomical localization. SPECT/CT has shown that there is a high metabolic lesion located posterior to the trachea at the T1 vertebral level, which guided surgery. For inconclusive cases, consider using 4D CT or PET/CT scans, for example with 18F-fluorocholine (14, 15).

### 3.3 Surgical challenges and strategies

Surgery for ectopic parathyroid tumors poses significant challenges (16): (i) the lesions are not where they are expected and (ii) their anatomy is complex being in close proximity to the recurrent laryngeal nerve, great vessels, thoracic duct and pleural apex. Based on our experience, we recommend:

1. Imaging navigation: The surgeon must personally review preoperative images to translate three-dimensional spatial information into an anatomical target. If the majority of the lesion is located above the jugular notch of the sternum, a surgical approach via a neck incision is entirely feasible. If the lesion is entirely located below the jugular notch of the sternum and cannot be accessed via a neck incision, a thoracoscopic approach may be necessary. In our case, initial negative exploration prompted re-review of images, leading to exploration posterior to the recurrent laryngeal nerve and into the mediastinum.
2. Systematic and expanded exploration: The order of common to rare is followed: dorsal thyroid, tracheoesophageal grooves, carotid sheath, retrosternal or superior thymic pole, retroesophageal space and superior mediastinum (partial sternotomy may be used if required) (17). We dissected the entire right recurrent laryngeal nerve and traced it toward the mediastinum, ultimately finding the tumor deep posterior to the nerve.
3. Meticulous dissection and protection: When examining deep ectopia, it is essential to adhere to the principle of meticulous membrane anatomy dissection and protection (18). Identify and protect the recurrent laryngeal nerve and major blood vessels. The tumor was adhered to the nerve; by exposing it first, injury was prevented by operating posterior to it. Extreme care should be taken in the vicinity of the pleural apex to avoid pneumothorax.
4. Intraoperative confirmation: Rapid frozen section pathology confirms parathyroid origin, verifying surgical success and avoiding incorrect resection or missed multifocal disease (19, 20).

### 3.4 Insights from this case

The PTH of the patient was found to be as high as 1920 pg/mL which indicates a large or highly functional adenoma. This is consistent with a 3 cm tumor nearby during intraoperative assessment. Sustained uncorrected high hormone levels can lead to adverse effects on bones and kidneys. This case underscores that for PHPT patients with markedly abnormal biochemical markers and negative or inconclusive initial imaging, aggressive use of SPECT/CT for precise localization is necessary. Surgeons must be psychologically and technically prepared for ectopic lesions. A negative exploration should be cross-checked with preoperative biochemical and functional imaging findings; otherwise, expanded exploration or staged thoracoscopic mediastinal exploration should be performed.

## 4 Conclusion

Ectopic parathyroid adenoma in the superior mediastinum is a common cause of surgical failure for PHPT. A successful management is achieved by accurate preoperative localization and accurate surgery. Comprehensive imaging assessment, particularly via 99mTc-MIBI SPECT/CT, offers a clear surgical “roadmap”. Successful surgery depends on the surgeon’s deep understanding of neck and superior mediastinal anatomy, meticulous preoperative planning, systematic intraoperative exploration, and refined microsurgical techniques. It is essential for a multidisciplinary approach on challenging cases.

## Data Availability

The original contributions presented in the study are included in the article/supplementary material. Further inquiries can be directed to the corresponding authors.

## References

[1] Alesina PF Donatini G Medas F . Editorial: Primary and secondary hyperparathyroidism: from etiology to treatment. Front Endocrinol (Lausanne). (2025) 16:1694239. doi: 10.3389/fendo.2025.1694239. PMID: 41347139 PMC12672265

[2] Lin YC Lee JC Wen CY Chiu WY . Hypercalcemia episodes caused by ectopic parathyroid adenoma and subsequent gastrointestinal stromal tumor: a case report and literature review. Front Oncol. (2025) 15:1720028. doi: 10.3389/fonc.2025.1720028. PMID: 41479802 PMC12753321

[3] Jung B Shin Y Lee S Hong N Nam SY Lee S . Ectopic parathyroid adenoma: successful localization with 18F-fluorocholine PET/CT and parathyroid venous sampling in two cases. BMC Endocr Disord. (2025) 25:289. doi: 10.1186/s12902-025-02131-8. PMID: 41430598 PMC12752095

[4] Kather FA Esfandiari NH Clines GA Janney CA Hughes DT Saberi S . Postparathyroidectomy bone density changes in patients with biochemically mild primary hyperparathyroidism. Endocr Pract. (2025) 19:S1530-891X(25)01333-3. doi: 10.1016/j.eprac.2025.11.019. PMID: 41421484

[5] Pepe J Minisola S Ettorre E Desideri G Cipriani C . Cardiovascular involvement in primary hyperparathyroidism. J Clin Endocrinol Metab. (2026) 111:603–14. doi: 10.1210/clinem/dgaf625. PMID: 41239190

[6] Khot R Dixe de Oliveira Santo I Shen L Itani M Sailer A Kirsch JD . US of the neck beyond the thyroid gland: parathyroid glands. Radiographics. (2025) 45:e240182. doi: 10.1148/rg.240182. PMID: 40440190

[7] Nakao E Kimura T Iwamoto H Tatsumi F Kaneto H . Primary hyperparathyroidism due to an ectopic parathyroid adenoma located in the posterior part of the esophagus. Cureus. (2025) 17:e86572. doi: 10.7759/cureus.86572. PMID: 40709121 PMC12286769

[8] He M Wiseman SM . Identifying the missing parathyroid: Remember to explore the carotid sheath. Am J Surg. (2025) 248:116430. doi: 10.1016/j.amjsurg.2025.116430. PMID: 40425431

[9] Sayeed S Joseph J Stanisce L Ramirez R Khorsandi A Brandwein-Weber M . Undescended ectopic parathyroid adenoma within ectopic cervical thymus in the carotid sheath. Head Neck. (2025) 47:E131–E3. doi: 10.1002/hed.28176. PMID: 40444454

[10] Salahaldin MM Zrineh A Keshek AA Karmi H Badran M Bourini M . Tertiary hyperparathyroidism with ectopic mediastinal parathyroid adenoma in a patient with chronic kidney disease and cardiovascular complications: a case report. BMC Endocr Disord. (2026) 26:30. doi: 10.1186/s12902-025-02160-3. PMID: 41484578 PMC12865936

[11] Gervasi R Piazzetta GL Soluri G Scigliano C Pelaia C Lobello N . A rare case of supernumerary and ectopic parathyroid adenoma in the parotid gland: diagnostic and surgical challenges. Diagn Pathol. (2025) 20:110. doi: 10.1186/s13000-025-01712-4. PMID: 41039631 PMC12492863

[12] Zhu R Yue N Hong Y Ye Q Han Z Luo Z . Ectopic parathyroid adenomas in primary hyperparathyroidism: anatomical classifications, localization strategies, and case series. Int J Surg. (2026) 112(4):9958–74. doi: 10.1097/JS9.0000000000004578. PMID: 41417934 PMC13105754

[13] Wakankar R Dharmashaktu Y Puri G Chumber S Dixit S Damle NA . Three different etiologies in three similar looking intrathyroidal nodules: role of Tc-99m sestaMIBI scintigraphy and SPECT-CT in a patient with primary hyperparathyroidism. Clin Nucl Med. (2025). doi: 10.1097/RLU.0000000000006142. PMID: 41428440

[14] Quak E Lasne-Cardon A Cavarec M Jegoux F Guery C Grellard JM . First-line [(18)F]F-choline PET/CT in primary hyperparathyroidism: a cost-effectiveness study from the diagnostic randomized APACH2 trial. Eur J Nucl Med Mol Imaging. (2026) 53:3291–6. doi: 10.1007/s00259-025-07728-8. PMID: 41491031 PMC13013383

[15] Klerkx S Decallonne B Meulemans J Boeckxstaens L Cockmartin L Vignero J . The role of 4DCT in the localization of parathyroid adenomas in primary hyperparathyroidism: a retrospective cohort study. Insights Imaging. (2025) 16:245. doi: 10.1186/s13244-025-02133-z. PMID: 41196525 PMC12592610

[16] Wei Y Zhao Z Wu J Cao S Yu N Cai W . Long-term outcomes and prognostic factors following microwave ablation vs surgical resection for primary hyperparathyroidism. Eur Radiol. (2026). doi: 10.1007/s00330-025-12254-0. PMID: 41492024

[17] Song JX Xie J Zhou JX Zhang YF Han RL Ye L . Approach to the patient with primary hyperparathyroidism in multiple endocrine neoplasia type 1. J Clin Endocrinol Metab. (2025) 110:3546–58. doi: 10.1210/clinem/dgaf486. PMID: 40878942

[18] Titu IM Silaghi CA Ciulic SA Teterea F Mlesnite M Palade E . Progress in the management of mediastinal ectopic parathyroid adenomas: the role of minimally invasive surgery. J Clin Med. (2025) 14:3020. doi: 10.3390/jcm14093020. PMID: 40364052 PMC12072443

[19] Block MA Frame B Kleerekoper M Rao DS . Surgical management of persistence or recurrence after subtotal parathyroidectomy for primary hyperparathyroidism. Am J Surg. (1979) 138:561–6. doi: 10.1016/0002-9610(79)90419-7. PMID: 484784

[20] Alnajmi RAY Ali DS Khan AA . Persistence and recurrence of primary hyperparathyroidism. Best Pract Res Clin Endocrinol Metab. (2025) 39:101986. doi: 10.1016/j.beem.2025.101986. PMID: 40074600

